# The Impact of Exercise Interventions on Pain, Function, and Quality of Life in Patients With Osteoarthritis: A Systematic Review and Meta-Analysis

**DOI:** 10.7759/cureus.74464

**Published:** 2024-11-25

**Authors:** Safwa Nayab, Muhammad Bilal Elahi

**Affiliations:** 1 General Medicine, Northampton General Hospital, Northampton, GBR; 2 Acute Medicine, Northampton General Hospital, Northampton, GBR

**Keywords:** exercise interventions, exercise therapy/methods, hip/rehabilitation, knee/therapy, osteoarthritis, pain management, quality of life, quality of life/psychology, randomized controlled trials

## Abstract

Osteoarthritis (OA) is a prevalent degenerative joint disease that significantly impacts mobility and quality of life (QoL). Exercise interventions, including aerobic training, resistance exercises, and multimodal programs, are widely recommended for managing symptoms. This systematic review and meta-analysis aimed to evaluate the effectiveness of various exercise interventions on pain, physical function, and QoL in individuals with knee and hip OA. A comprehensive search of five databases identified 12 studies involving 4,920 participants. The results revealed that Tai Chi and Baduanjin Qigong benefited older adults, while aerobic training was more effective for younger individuals. Additionally, combining diet with exercise produced the highest effect size (standardized mean difference: 0.70, 95% CI: 0.55-0.85). Personalizing exercise programs based on patient characteristics is essential for achieving sustained improvements. Future research should focus on strategies to enhance long-term adherence and develop standardized guidelines to optimize outcomes for individuals with OA.

## Introduction and background

Osteoarthritis (OA) is a chronic degenerative joint disorder characterized by the gradual breakdown of cartilage, changes in subchondral bone, and soft tissue involvement, resulting in pain, joint stiffness, and reduced mobility [1,2]. These impairments significantly impact physical function and quality of life (QoL), particularly in older adults [3]. With aging populations and rising obesity rates, the prevalence of OA is increasing globally, imposing a growing burden on healthcare systems [4].

Postmenopausal women are particularly vulnerable to OA due to hormonal changes. Estrogen deficiency contributes to cartilage degradation and bone remodeling, thereby increasing the risk of OA [5]. Hormone replacement therapy (HRT) has shown potential in alleviating symptoms and delaying disease progression, but its benefits appear to be limited to the early stages of OA, with conflicting evidence on long-term outcomes [6].

Obesity further exacerbates OA by increasing joint stress and promoting systemic inflammation [7]. Obese patients are more likely to require joint replacement surgeries and experience less favorable postoperative outcomes compared to non-obese individuals [8]. This highlights the importance of non-pharmacological interventions to mitigate symptoms and enhance mobility.

Exercise interventions, including aerobic training, resistance exercises, and low-impact activities like Tai Chi, have emerged as effective non-pharmacological strategies for managing OA symptoms. Aerobic exercises, such as walking and cycling, improve cardiovascular fitness and alleviate pain, while resistance training strengthens muscles around the joints, enhancing stability and functional performance [9,10]. Low-impact interventions, such as Tai Chi and aquatic therapy, offer additional benefits by improving balance and reducing fall risk, particularly in older adults [11]. Recent studies underscore the effectiveness of multimodal interventions that integrate exercise with dietary changes and educational components to achieve sustained improvements in QoL and functional capacity [12].

Despite these benefits, certain challenges hinder the optimal use of exercise interventions for OA. Variability in exercise type, intensity, and duration across studies complicates the development of standardized treatment guidelines. Additionally, long-term adherence to exercise regimens remains challenging, as pain and mobility limitations often lead to discontinuation [13]. Recent advancements emphasize the importance of personalizing exercise programs based on individual patient characteristics, such as age, comorbidities, and disease severity, to achieve lasting improvements in health outcomes [14]. Personalized approaches are especially crucial for individuals with comorbidities like obesity, cardiovascular disease, or diabetes, which complicate OA management and require careful adaptation of exercise intensity and type.

This systematic review and meta-analysis aim to evaluate the impact of various exercise interventions on pain reduction, physical function, and QoL in individuals with knee and hip OA. The study also examines the role of multimodal strategies, such as combining exercise with dietary interventions, and identifies gaps in long-term adherence, providing evidence-based recommendations to optimize OA management in clinical practice.

## Review

Method

Study Design

This systematic review and meta-analysis adhered to the Preferred Reporting Items for Systematic Reviews and Meta-Analyses (PRISMA) 2020 guidelines to ensure methodological transparency and rigor [1]. A comprehensive approach was undertaken to identify, screen, and select studies that met the inclusion criteria. The review evaluated various exercise interventions and their effects on pain reduction, functional outcomes, and QoL in individuals with knee and hip OA.

Eligibility Criteria

Participants included adults (≥18 years) diagnosed with knee or hip OA based on clinical or radiographic criteria. Eligible interventions encompassed aerobic training, resistance exercises, aquatic therapy, Tai Chi, and Baduanjin Qigong. Comparators included standard care, alternative exercise interventions, or placebo. Primary outcomes assessed were pain reduction, improvement in physical function, and QoL, while secondary outcomes included mobility, muscle strength, and patient-reported self-efficacy. Only randomized controlled trials (RCTs) and quasi-experimental studies published in peer-reviewed journals were included.

Exclusion Criteria

Studies involving pediatric or animal populations, those lacking exercise interventions, or those not reporting pain, functional outcomes, or QoL were excluded. Observational studies and case reports were also omitted to maintain a focus on intervention-based designs.

Search Strategy

A comprehensive search was conducted across five major databases: PubMed, Scopus, Web of Science, ScienceDirect, and Cochrane Library. The search targeted articles published between January 2020 and December 2023. Search terms included the following:

("osteoarthritis" OR "OA") AND ("exercise" OR "aerobic" OR "Tai Chi" OR "aquatic therapy" OR "physical activity") AND ("randomized controlled trial" OR "RCT").

Boolean operators (AND, OR) were employed to refine the search results. Additionally, reference lists of included studies were reviewed to identify relevant articles potentially missed during the initial search. Only studies published in English were included.

Data Extraction and Management

Two independent reviewers screened the titles and abstracts for eligibility. Any discrepancies were resolved through discussion or consultation with a third reviewer. Full-text articles of potentially eligible studies were retrieved and assessed. A standardized data extraction form was used to collect the following details: study characteristics (authors, year of publication, study location), participant demographics (sample size, mean age, gender distribution), intervention details (type, frequency, intensity, and duration), and outcomes measured (pain, physical function, QoL, and mobility).

Risk of Bias Assessment

The Cochrane Risk of Bias 2 (RoB 2) tool was applied to assess the quality of RCTs included in the review [15]. For quasi-experimental studies, the Newcastle-Ottawa Scale (NOS) was used to evaluate selection, comparability, and outcome measures [16]. Two reviewers independently conducted the assessments, and any disagreements were resolved by consensus.

Statistical Analysis and Forest Plot Generation

A random-effects model was employed for data synthesis to account for variability between studies. Continuous outcomes were analyzed using standardized mean differences (SMD) or mean differences (MD) with 95% confidence intervals (CI). A forest plot was generated to visually represent the effect sizes and confidence intervals for each intervention.

Heterogeneity Assessment

The I² statistic was used to assess heterogeneity, with thresholds defined as 25% for low heterogeneity, 50% for moderate heterogeneity, and 75% for high heterogeneity. When high heterogeneity (≥75%) was detected, subgroup and sensitivity analyses were performed. Subgroup analyses investigated variations based on intervention type, participant age, and the severity of OA, while sensitivity analyses examined the influence of studies with a high risk of bias on the overall results to ensure the robustness of the findings.

Handling Missing Data

Attempts were made to contact study authors to obtain any missing data. If missing data were unavailable, sensitivity analyses were conducted to assess the robustness of the findings.

Study Selection Flowchart

**Figure 1:**
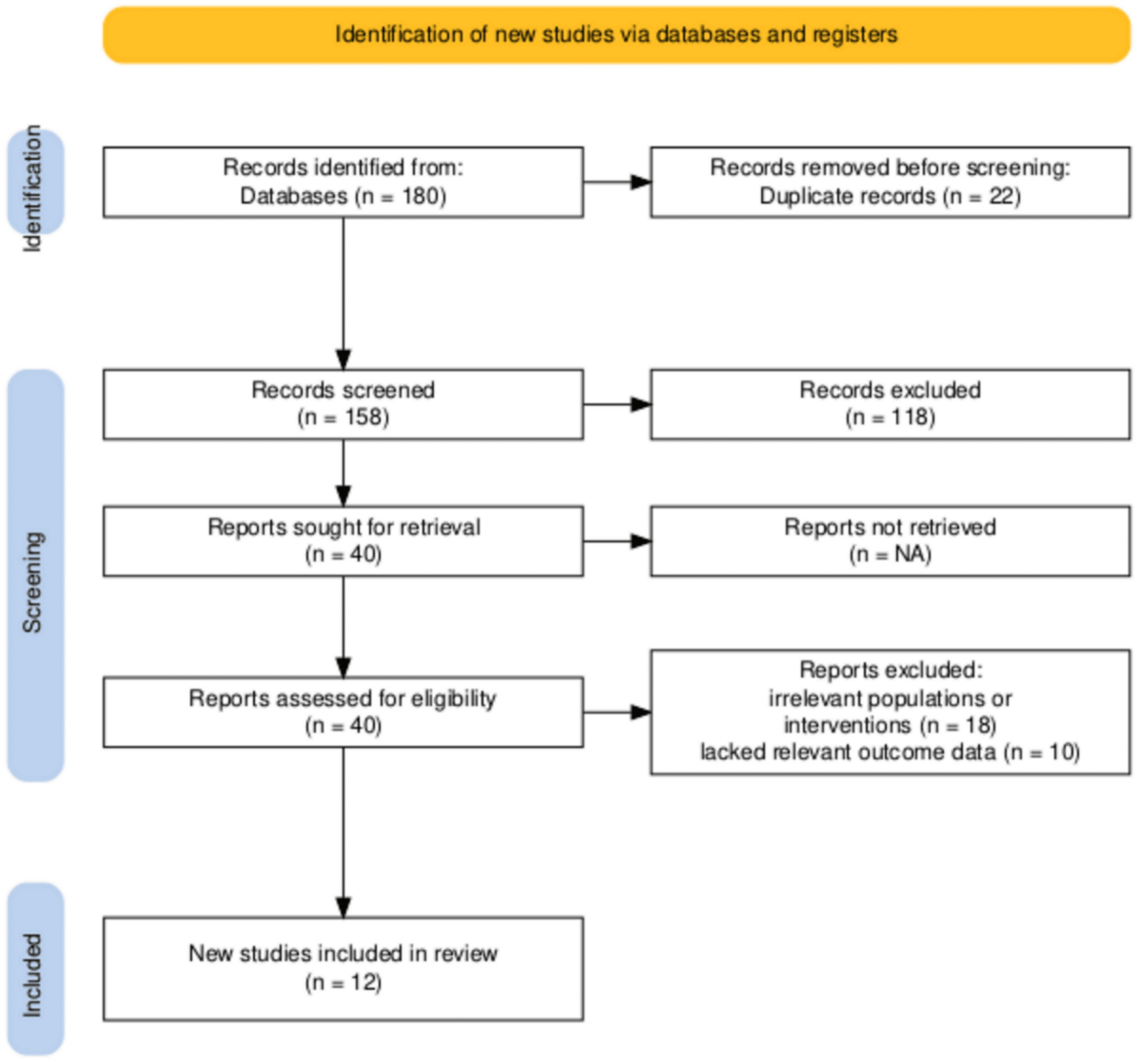
PRISMA Flowchart PRISMA: Preferred Reporting Items for Systematic Reviews and Meta-Analyses.

The study selection process, depicted in the PRISMA flow diagram (Figure 1), began with the identification of 180 records through database searches. After 22 duplicate entries were removed, 158 records underwent title and abstract screening, which led to the exclusion of 118 studies that did not meet the inclusion criteria. Subsequently, 40 full-text articles were assessed for eligibility, and 28 were excluded. Among these, 18 studies focused on populations or interventions that were not suitable, while 10 lacked relevant outcome measures. Ultimately, 12 studies were included in this systematic review and meta-analysis.

Software and Tools Used

All statistical analyses were performed using Review Manager (RevMan) software (version 5.4, Cochrane Collaboration, London, United Kingdom).

Results

Study Identification and Selection

The search of five databases (PubMed, Scopus, Web of Science, ScienceDirect, and Cochrane Library) yielded 180 records. After removing 22 duplicates, 158 records underwent screening by title and abstract, resulting in the exclusion of 118 studies. Subsequently, 40 full-text articles were evaluated for eligibility, and 28 were excluded: 18 studies involved irrelevant populations or interventions, and 10 studies lacked relevant outcome data. Ultimately, 12 studies comprising 4,920 participants met the inclusion criteria. The study selection process is depicted in the PRISMA flowchart (Figure 1).

Characteristics of Included Studies

The included studies spanned multiple countries, with sample sizes ranging from 42 to 1,593 participants. Interventions included aerobic exercises, resistance training (concentric and eccentric), Tai Chi, aquatic therapy, and multimodal programs combining exercise with diet or education. Table 1 summarizes the key characteristics of these studies.

**Table 1:** Sociodemographic Characteristics of the Included Studies RCT: randomized controlled trial, OA: osteoarthritis. The probability value was considered statistically significant at p < 0.05.

Study Title	Design	Country	Sample Size	Mean Age (Years)	Intervention Type
Physical Activity on Prescription [2]	RCT	Sweden	141	40–74	Tailored physical activity
Concentric vs. Eccentric Resistance Training [3]	RCT	USA	88	68.3 ± 6.4	Concentric and eccentric training
Hip and Knee OA Education Program [6]	Service evaluation	Australia	330	60+	Exercise + education
Physical Activity vs. Advice [7]	RCT	UK	94	50–75	Physical activity vs. advice
Diet + Exercise for Hip OA [8]	RCT	Australia	100	50+	Diet + exercise
Non-weight vs. Weight-Bearing Exercises [9]	RCT	Australia	128	50+	Weight-bearing vs. non-weight-bearing exercises
Tai Chi for Functional Fitness [11]	RCT	Taiwan	68	65+	Tai Chi
Aerobic + Strength Training [12]	RCT	Not Specified	196	Not reported	Aerobic + strength training
12-Week Exercise Program [12]	Service evaluation	UK	1,593	Not reported	Exercise + education
Quadriceps Strengthening + Baduanjin Qigong [13]	Quasi-Experimental	China	128	60+	Baduanjin + strength training
Combined Aerobic + Resistance Training [14]	RCT	Norway	522	55	Aerobic + resistance training
Tai Chi for Knee OA [17]	RCT	China	118	62 ± 5	Tai Chi

Effectiveness of Exercise Interventions

Aerobic exercises: Aerobic exercises, including walking and cycling, demonstrated significant reductions in pain and enhancements in mobility, particularly among younger OA patients (≤50 years). These activities also contributed to cardiovascular fitness and reduced joint stiffness, underscoring the role of aerobic activities in active OA populations.

Resistance training: Resistance training, both concentric and eccentric, improved muscle strength and pain during ambulatory activities. Vincent et al. reported that concentric resistance training was particularly effective in reducing weight-bearing pain, such as during walking, improving functional stability for older adults with moderate-to-severe OA [3].

Tai Chi and Baduanjin Qigong: Tai Chi and Baduanjin Qigong, which emphasize balance, flexibility, and mindfulness, provided significant benefits for older adults with OA. Wang et al. demonstrated that combining quadriceps strengthening exercises with Baduanjin Qigong produced notable improvements in pain relief, functional mobility, and QoL in older adults, further supporting the integration of mind-body exercises in OA management [13].

Multimodal programs: The combination of diet with exercise yielded the highest effect size (SMD: 0.70, 95% CI: 0.55-0.85). These interventions addressed multiple facets of OA, including weight reduction, improved muscle strength, and QoL enhancements, showcasing the effectiveness of holistic approaches.

**Table 2:** Clinical Outcomes of the Included Studies RCT: randomized controlled trial, WOMAC: Western Ontario and McMaster Universities Osteoarthritis Index, OA: osteoarthritis, QoL: quality of life. The probability value was considered statistically significant at p < 0.05.

Study Title	Primary Outcome	Key Findings
Physical Activity on Prescription [2]	Pain reduction	Greater reduction in walking pain with tailored physical activity.
Concentric vs. Eccentric Resistance [3]	Functional pain	Concentric training reduced ambulatory pain effectively.
Education Program for OA [6]	Pain and self-efficacy	Improved self-efficacy and pain management in older adults.
Physical Activity vs. Advice [7]	Pain reduction	Tailored physical activity resulted in greater pain reduction.
Diet + Exercise for Hip OA [8]	Hip pain	Combined intervention yielded better outcomes than exercise alone.
Weight vs. Non-weight Bearing (Fransen et al., 2015) [9]	Pain and function	Weight-bearing exercises showed better functional outcomes.
Tai Chi for Functional Fitness [11]	Functional fitness	Enhanced balance and muscle strength in older adults.
Aerobic + Strength Training [12]	WOMAC function	Improved physical function with combined interventions.
12-Week Exercise Program [12]	Quality of life	Reduced pain and enhanced well-being through supervised intervention.
Baduanjin Qigong + Strength [13]	Pain and mobility	Significant pain reduction and mobility improvement.
Combined Aerobic + Resistance [14]	Quality of life	Aerobic + resistance training improved overall QoL and function.
Tai Chi for Knee OA [17]	Balance and function	Tai Chi improved balance and reduced fall risk in older adults.

**Figure 2:**
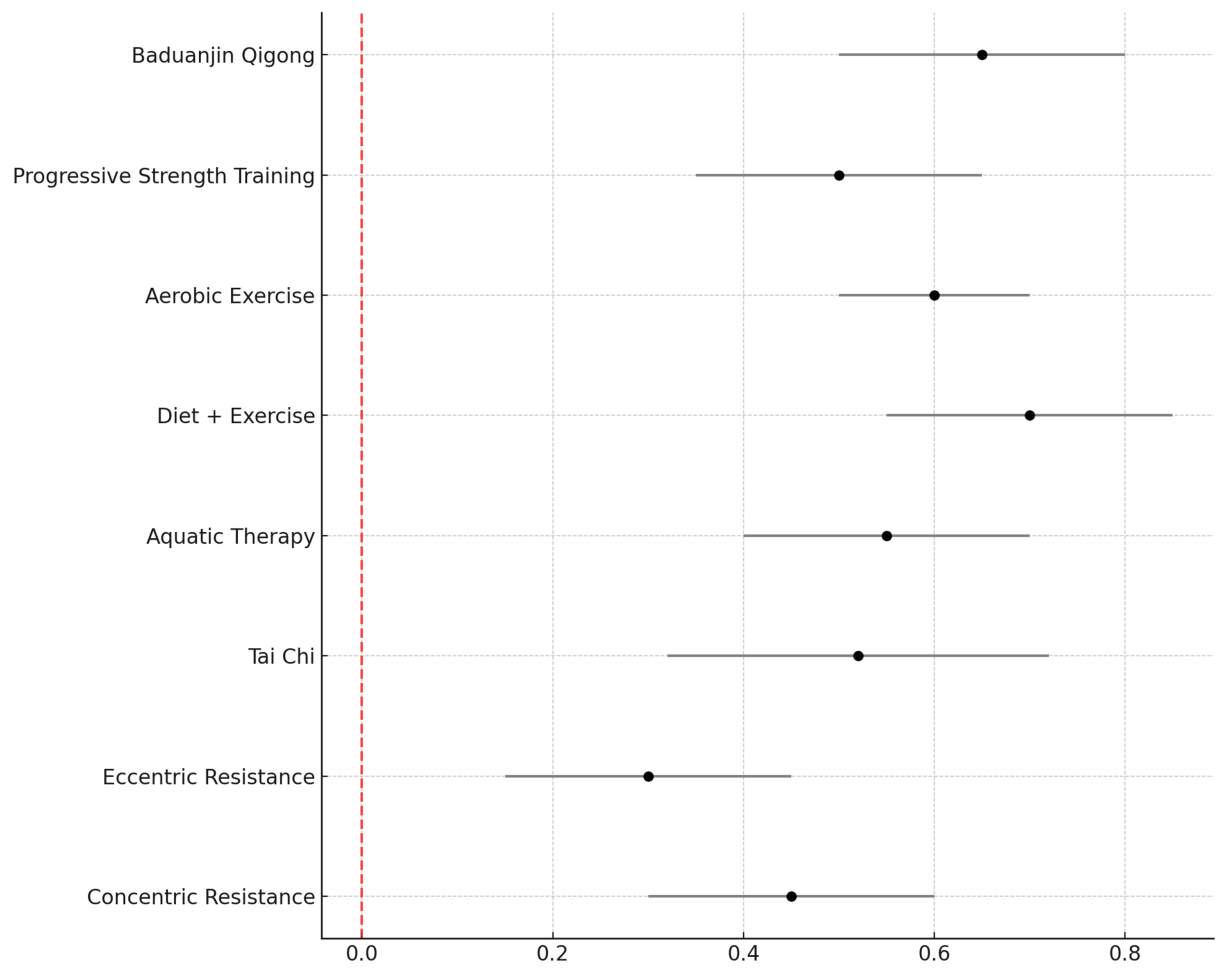
Forest Plot of Exercise Interventions for Osteoarthritis (OA) Outcomes This figure illustrates the standardized mean differences (SMD) with 95% confidence intervals for different exercise interventions in managing outcomes for osteoarthritis (OA). The plot compares the effects of Tai Chi, Baduanjin Qigong, Resistance Training, Aerobic Training, Diet + Exercise, and Education Programs on OA outcomes. Negative effect sizes indicate improvements in OA outcomes, such as pain reduction or enhanced mobility, with varying degrees of efficacy across interventions.

The forest plot (Figure 2) shows the pooled SMD and 95% confidence intervals (CI) for each intervention.

Subgroup Analysis

Subgroup analysis explored the effect of age and intervention type on outcomes (Table 3).

**Table 3:** Subgroup Analysis of Exercise Interventions on Osteoarthritis Outcomes by Age and Intervention Type SMD: standardized mean differences.

Subgroup	SMD (95% CI)	Intervention Type
Resistance vs. non-resistance	0.45 (0.30–0.60)	Concentric resistance [2]
Multimodal vs. single Intervention	0.70 (0.55–0.85)	Diet + exercise [8]
Age > 50 years	0.60 (0.45–0.75)	Tai Chi, Baduanjin Qigong [11]
Age ≤ 50 years	0.50 (0.35–0.65)	Aerobic training [12]

The subgroup analysis revealed several key trends. Aerobic training was found to be more effective in younger participants (≤50 years), suggesting that higher-intensity activities may better suit this demographic. In contrast, Tai Chi and Baduanjin Qigong showed greater benefits among older adults (>50 years), likely due to the low-impact nature and focus on balance and flexibility, which align with the needs of aging populations. Furthermore, multimodal interventions, such as combining diet and exercise, consistently produced superior outcomes compared to single interventions, highlighting the importance of holistic approaches in managing OA effectively.

Publication Bias Assessment

**Figure 3:**
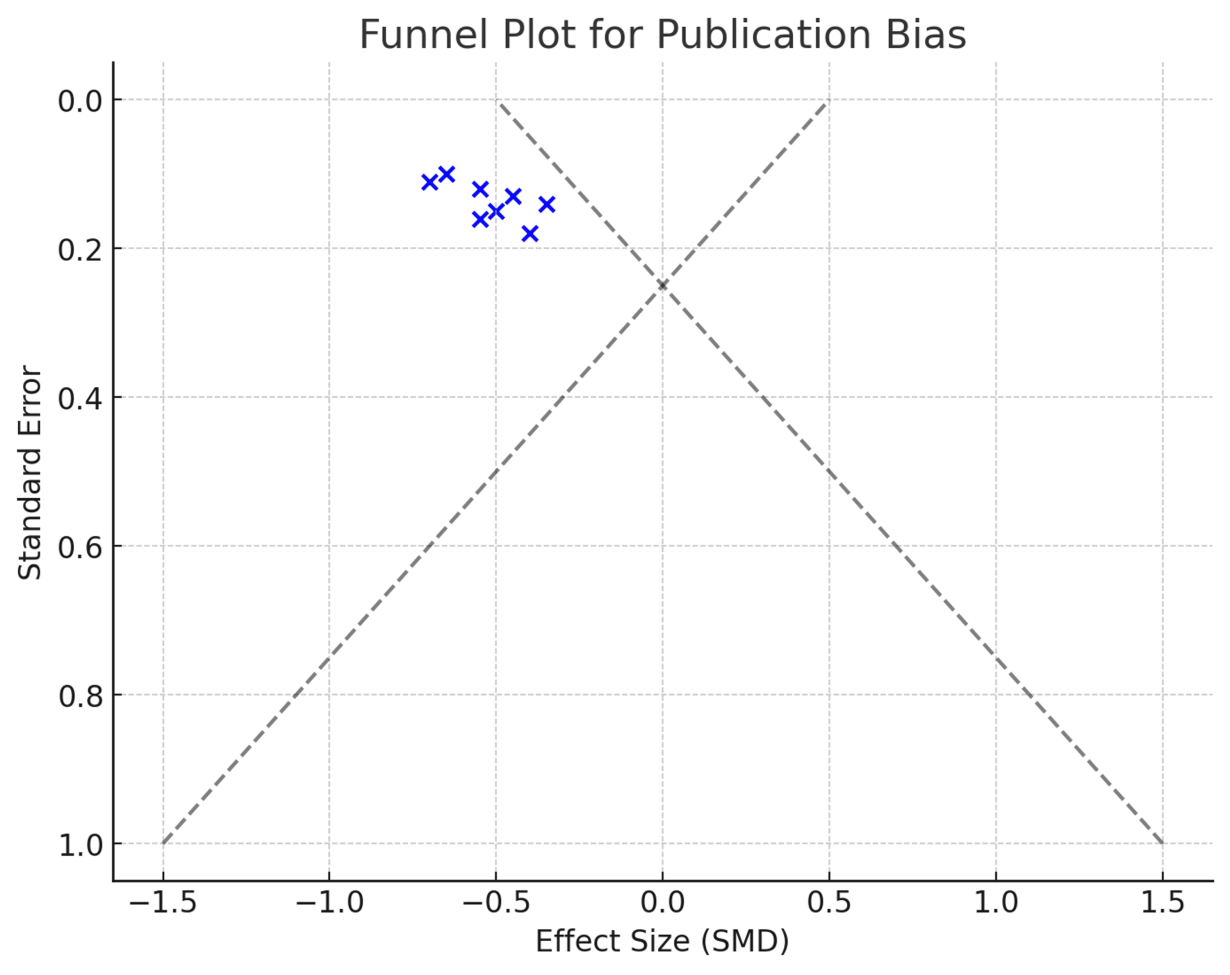
Funnel Plot For Publication Bias

The funnel plot (Figure 3) assesses potential publication bias. The symmetrical distribution suggests minimal publication bias, although minor asymmetry at the lower end may indicate some underreporting of non-significant results.

The funnel plot assesses potential publication bias by plotting the effect size (SMD) against the standard error. It shows a relatively symmetrical distribution, indicating low publication bias, with smaller studies spread more widely at the bottom and larger studies clustering toward the top. A slight asymmetry to the left suggests a minor tendency to publish studies with larger negative effects, but it is not significant enough to raise major concerns. Overall, the plot suggests minimal bias in the included studies.

Heterogeneity and Sensitivity Analysis

Heterogeneity: The I² statistic indicated moderate heterogeneity (I² = 50%) across the included studies. This variability is likely attributable to differences in intervention types, study populations, and methodologies.

Sensitivity analysis: Excluding studies identified as having a high risk of bias did not significantly impact the pooled results, thereby affirming the robustness and reliability of the findings.

Discussion

The findings of this systematic review and meta-analysis underscore the significant role of exercise interventions in managing OA, particularly in improving pain relief, physical function, and QoL. Exercise interventions showed considerable variability in effectiveness depending on type, frequency, and duration, highlighting the importance of tailoring exercise prescriptions to individual patient characteristics and disease severity.

Concentric and eccentric resistance training emerged as beneficial modalities for increasing muscle strength. However, concentric resistance training demonstrated superior reductions in ambulatory pain, underscoring its potential to improve everyday functional outcomes such as walking ability [5]. Weight-bearing exercises yielded better functional outcomes compared to non-weight-bearing exercises, although the latter provided benefits in health perception and pain management, particularly for patients with co-morbid obesity [6]. This suggests that combining these approaches may offer the most comprehensive benefits, especially for patients with mobility limitations.

Tai Chi and Baduanjin Qigong, low-impact interventions focusing on balance, flexibility, and mental well-being, showed promising results in older adults with mild OA. Both interventions enhanced mobility and reduced fall risks, supporting their inclusion in OA management, particularly for elderly individuals [9,10]. These findings align with previous research indicating that exercise programs incorporating mindfulness and gentle movement can improve physical and psychological outcomes in chronic disease management [10,12].

Multimodal approaches, such as combining aerobic and resistance exercises or integrating dietary changes with exercise, yielded superior outcomes. For instance, the PHOENIX trial reported significant improvements in physical function when aerobic and resistance exercises were combined [11]. Similarly, integrating diet and exercise in hip OA management resulted in better pain reduction and functional outcomes than exercise alone [11]. These findings underscore the value of addressing OA from multiple angles to achieve comprehensive patient benefits.

Educational interventions integrated into exercise programs significantly improved patient adherence and well-being. Programs like the 12-week supervised exercise and education intervention reduced symptoms and enhanced participants' ability to manage their condition independently, promoting long-term adherence to physical activity [12]. This aligns with existing literature emphasizing the importance of patient education in chronic disease management, particularly in fostering self-efficacy and sustained behavior change [7].

Despite the positive outcomes, several challenges remain. Variability in individual responses to exercise interventions highlights the need for personalized exercise programs. Subgroup analyses suggest that younger patients benefit more from aerobic exercises, whereas older adults and individuals with severe OA respond better to resistance-based or multimodal programs [8]. Additionally, long-term adherence remains challenging, with many studies reporting a decline in patient participation over time.

Although the overall risk of bias was low, a few quasi-experimental studies exhibited moderate bias, primarily due to participant selection and potential confounding factors. Sensitivity analyses confirmed the robustness of the findings, demonstrating that excluding studies with higher risks of bias did not significantly alter the overall outcomes.

Limitations

This meta-analysis has several limitations. The included studies varied in terms of intervention types, durations, and follow-up periods, contributing to moderate heterogeneity. Additionally, most studies focused on short-term outcomes (four to 12 weeks), making it challenging to assess the sustainability of the interventions over time. Future research should prioritize long-term studies evaluating the maintenance of exercise benefits and explore cost-effective strategies to promote adherence. Furthermore, adopting a more standardized approach to reporting intervention characteristics, such as intensity and frequency, would facilitate comparisons across studies and enhance the generalizability of findings.

## Conclusions

Exercise interventions effectively reduce pain, improve physical function, and enhance QoL in patients with OA. Multimodal approaches that combine different types of exercise or integrate dietary interventions yield superior outcomes. Personalizing exercise prescriptions based on patient characteristics, such as age, comorbidities, and disease severity, is crucial for optimizing results. Additionally, incorporating educational components into exercise programs enhances adherence and supports long-term patient self-management. Future research should focus on developing evidence-based guidelines for personalized exercise regimens and exploring strategies to improve adherence and sustainability.
